# Evaluation of Sterilization/Disinfection Methods of Fibrous Polyurethane Scaffolds Designed for Tissue Engineering Applications

**DOI:** 10.3390/ijms21218092

**Published:** 2020-10-30

**Authors:** Iwona Łopianiak, Beata A. Butruk-Raszeja

**Affiliations:** Faculty of Chemical and Process Engineering, Warsaw University of Technology, Waryńskiego 1, 00-645 Warsaw, Poland; Beata.Raszeja@pw.edu.pl

**Keywords:** sterilization, solution blow spinning, polyurethane, fibers, ethanol disinfection, antimicrobial solution, UV disinfection, gamma sterilization, electron beam sterilization

## Abstract

Sterilization of a material carries the risk of unwanted changes in physical and chemical structure. The choice of method is a challenge—the process must be efficient, without significantly changing the properties of the material. In the presented studies, we analyzed the effect of selected sterilization/disinfection techniques on the properties of nanofibrous polyurethane biomaterial. Both radiation techniques (UV, gamma, e-beam) and 20 minutes’ contact with 70% EtOH were shown not to achieve 100% sterilization efficiency. The agar diffusion test showed higher sterilization efficiency when using an antimicrobial solution (AMS). At the same time, none of the analyzed techniques significantly altered the morphology and distribution of fiber diameters. EtOH and e-beam sterilization resulted in a significant reduction in material porosity together with an increase in the Young’s modulus. Similarly, AMS sterilization increased the value of Young’s modulus. In most cases, the viability of cells cultured in contact with the sterilized materials was not affected by the sterilization process. Only for UV sterilization, cell viability was significantly lower and reached about 70% of control after 72 h of culture.

## 1. Introduction

An ideal scaffold should have the desired properties and a positive effect on cells and tissues [1]. Sterilization is a process that allows eliminating all forms of living organisms, including bacteria, viruses, and yeasts, which can be found on materials. The selection of an effective sterilization method is extremely important in order to avoid contamination of the samples, as well as undesirable changes in physical and chemical properties of the sterilized material [2,3]. The effectiveness of the sterilization method depends on the type and number of microorganisms present on the sterilized material [4] and properties of the sterilized scaffolds. Therefore, there is a need to choose a sterilization technique appropriate to the character of the material.

Techniques that are the most commonly used for biomaterials sterilization include chemical treatment (ethanol, ethylene oxide), antibiotic treatment, irradiation techniques (ultraviolet irradiation, gamma and electron beam irradiation), and heat treatment. Each of the sterilization methods has its advantages and limitations.

Chemical sterilization with ethylene oxide (EO) is a very effective method that can be performed at a low temperature. Application of low temperature makes EO sterilization the only acceptable method for temperature-sensitive biomaterials. In addition, it is a cheap and easily available method. However, residues of EO can cause changes in surface morphology and can be toxic to cells after implantation. Therefore, long aeration of sterilized materials after the sterilization process is necessary to get rid of all harmful EO [5,6,7,8].

Disinfection with ethanol solution is often used for fibrous materials. The mechanism of alcohol disinfection is based on dissolution of cell membrane lipids, denaturation of proteins, and cellular dehydration. The most commonly used solution is a 60–80% *v/v* ethanol solution [9,10]. There are many publications in which fibrous materials made by electrospinning have been disinfected with alcohol. It has been observed that, despite the intense washing with phosphate-buffered saline, ethanol solution remains inside the porous material [2,11,12].

Heat treatment including steam and dry heat sterilization is an effective, simple and fast sterilization method. It’s nontoxic for sterilized materials and for the environment. However, long exposure to high temperatures may damage most biodegradable polymers [4,6].

Irradiation sterilization/disinfection techniques include gamma, electron beam (e-beam), and ultraviolet (UV) irradiation. Gamma irradiation is a simple, effective, high penetrating sterilization methods. As a source of gamma rays, 60 Cobalt is most commonly used [3]. The effect of gamma radiation on the sterilized material depends on the type of material and its initial mass. This type of sterilization also changes material’s morphology together with mechanical and chemical properties [13,14]. Electron beam (e-beam) irradiation uses a high-energy electrons to sterilize materials. This treatment results in the disruption of DNA chains and degradation of chemical and molecular bonds inside cells. E-beam is distinguished by low penetration but, in contrast to gamma irradiation, this method requires high energy [2,4]. Disinfection with UV irradiation allows killing all microorganisms present only on the surface of the sterilized material. Thus, for disinfection to be effective, the material must be flat enough. It is certainly not a method of choice in cases of highly porous scaffolds. Most often, UV disinfection is used in combination with other methods. It requires quite long exposure time and may affect chemical and physical material properties [15].

Antimicrobial solutions consist of various antibiotics and/or antimycotics. The most commonly used are penicillin, gentamicin, vancomycin, ciprofloxacin, and amphotericin, cefoxitin, lincomycin. The exact composition depends on the type of sterilized material—for high sterilization efficiency it is necessary to choose the right mix of antibiotics depending on the microorganisms present on the material [2,12,16,17].

In this study fibrous materials were produced by solution blow spinning method. Solution blow spinning (SBS) is a simple and well-known method, which is distinguished by lower equipment requirements, high efficiency, and relatively low production costs compared to other fiber production methods, e.g., electrospinning [18,19]. It was decided to use medical-grade biodegradable polyurethane as a polymer of choice. This choice was made due to the relatively low thrombogenicity of this polymer. Such polyurethane fibrous materials could be a promising material for the development of vascular prostheses.

Fibrous materials have various other biomedical applications due to the possibility to control their morphology and, as a result, mechanical properties. They can be used as drug carriers for controlled drug delivery systems through their large surface area to volume ratio providing smooth drug release [20,21]. A wide range of fiber diameters and various mechanical properties provide its application as scaffolds for tissue regeneration (e.g., blood vessels [22], bones [23], nerves [24], and cartilages [25]). In addition, due to their porous structure fibrous materials can be applied as dressings for wound healing [26].

The aim of this study was to compare various sterilization/disinfection techniques applied for fibrous polyurethane scaffolds in the terms of their effectiveness, impact on scaffolds’ morphology, chemical composition, mechanical properties, and viability of cells contacted with sterilized materials. Fibrous biomaterials analyzed in the study were produced from polyurethane solution by solution blow spinning method. Five different sterilization/disinfection techniques were analyzed: antimicrobial solution, ethanol solution, UV irradiation, gamma irradiation, and e-beam irradiation.

## 2. Results

### 2.1. Evaluation of Sterilization Efficiency

#### 2.1.1. Visual Inspection

As shown in Table 1 no visible changes in color and material structure were observed after sterilization for each sterilization technique. After 72 h of cell culture on gamma-irradiated materials, color change from white to yellow was observed.

#### 2.1.2. Contact with Medium

As shown in Table 1, during the seven days of incubation at 37 °C, at 5% CO_2_ humified atmosphere, no changes (contamination) in culture medium were observed in any of the tested samples. Surprisingly, there were also no signs of contamination in medium contacted with unsterile (control) material.

#### 2.1.3. Agar Diffusion Method

The results of the agar diffusion test are presented in Table 1. After 24 h of incubation at 37 °C, no visible changes to any Petri dishes were observed. After 48 h of incubation at 37 °C, contamination was observed in one Petri dish with unsterilized material (positive control) and one Petri dish with gamma-irradiated material. After 72 h of incubation at 37 °C the contamination was observed in three Petri dishes with unsterilized material (positive control), three Petri dishes with gamma-irradiated material, one Petri dish with UV-irradiated material, and two Petri dishes with e-beam-irradiated material. After seven days’ incubation at 37 °C contamination was observed in three Petri dishes with unsterilized material (positive control), three Petri dishes with gamma-irradiated material, two Petri dishes with UV-irradiated material, three Petri dishes with e-beam-irradiated material, and one Petri dish with EtOH-treated material.

To summarize, after seven days of incubation at 37 °C the contamination was observed in three out of three samples with unsterilized material (positive control), three out of three samples with gamma-irradiated material, two out of three samples with UV-irradiated material, three out of three samples with e-beam irradiated material, and one out of three samples with EtOH treated material. In all samples sterilized with AMS (both variants: 1 h and 24 h) no contamination was observed during the seven days of incubation at 37 °C.

### 2.2. Material Characterization

#### 2.2.1. Surface Morphology

**Figure 1:**
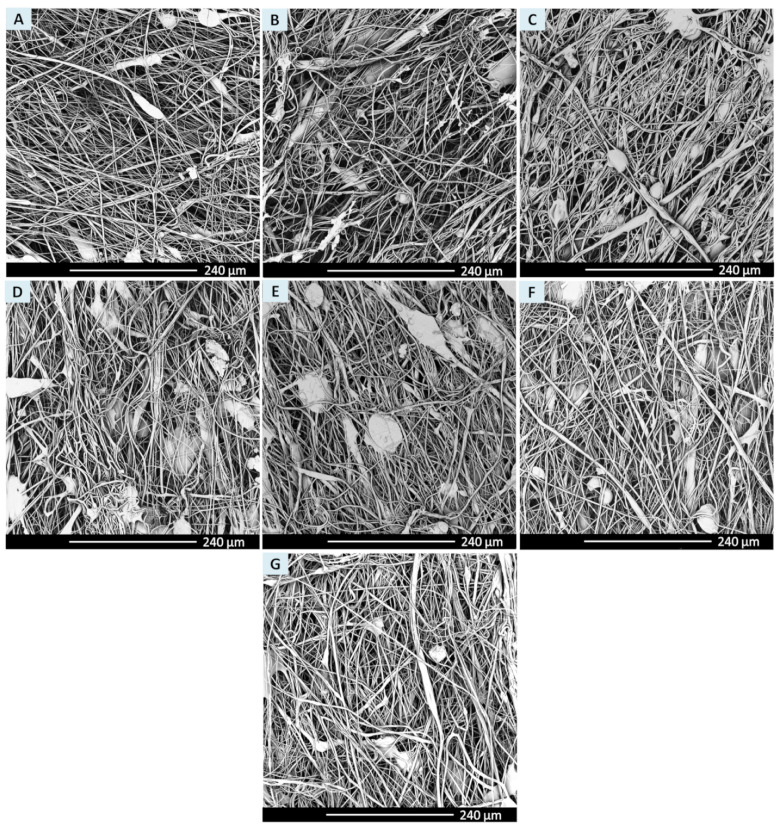
Sterilized materials scanning electron microscopy (SEM) images in magnification ×600 (**A**) unsterile material, (**B**) antimicrobial solution (AMS) for 1 h, (**C**) AMS for 24 h, (**D**) UV irradiation, (**E**) EtOH, (**F**) gamma irradiation, (**G**) e-beam irradiation.

SEM images of all investigated surfaces are presented in Figure 1. Morphology observation revealed the presence of unaligned fibers with local defects such as stains, swellings, and spindles which are common for this type of material. Fibers were relaxed (not stretched), which is a result of the type of polymer. ChronoFlex C75D polymer is a rigid material according to the Shore scale. There were no damaged or broken fibers observed for any of the tested material variants. Also, the number of local defects was similar for all tested variants.

**Figure 2:**
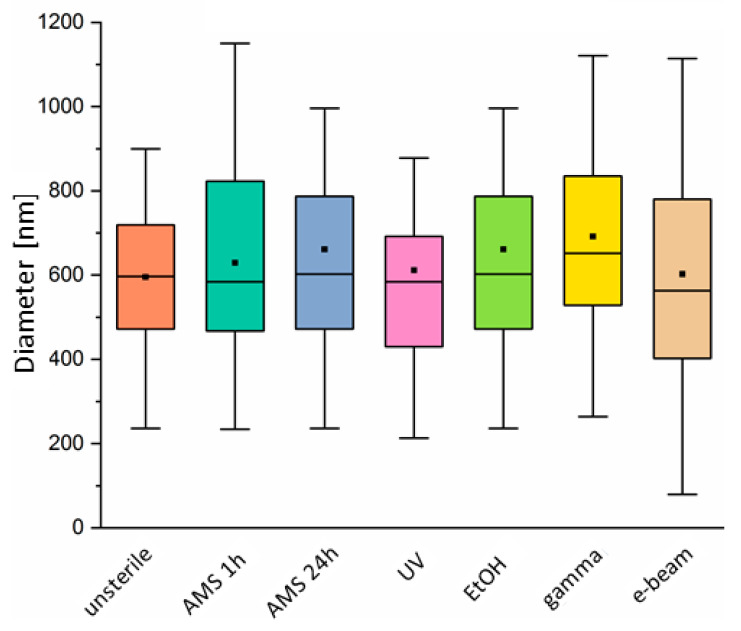
Distribution of fiber diameters before and after sterilization.

The range of fiber diameters for each sterilization type is shown in Figure 2. No significant changes in diameters were noticed before and after the sterilization process. In most cases, the diameter range has been shifted to higher values, which is represented by an increase in the mean value of the fiber diameter. Only in the case of e-beam sterilization, the lower diameter range has moved towards lower values compared to the control material. The average fiber diameter for unsterile (control) material equaled to 595 nm. The maximum change in fiber diameter was observed for material treated with EtOH and gamma rays. In both cases, the average fiber diameter increased about 15–16% compared to the control. AMS 24 h sterilization resulted in an increase in the average fiber diameter of about 11%. For materials sterilized with AMS for 1 h, UV and e-beam irradiation change in fiber diameter was less than 10%.

**Figure 3:**
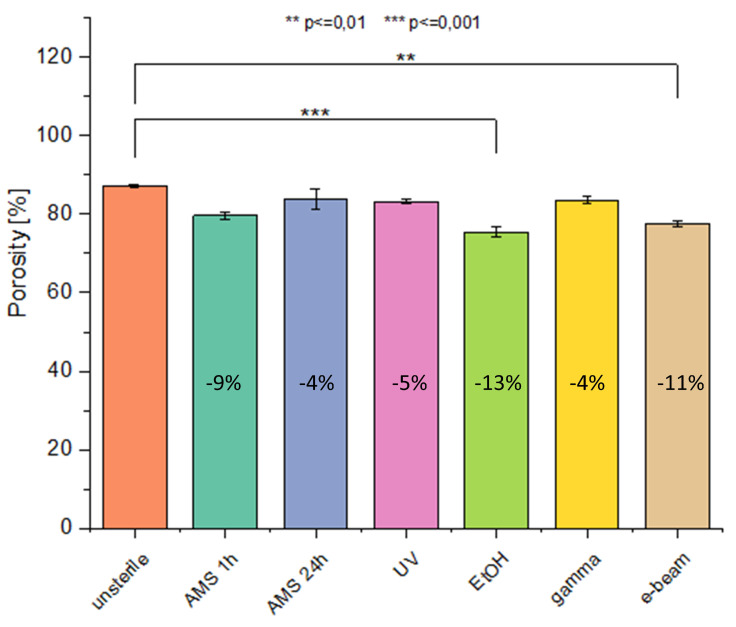
Materials’ porosity before and after sterilization, mean value (MV) ± SD, *n* = 3.

The porosity of the material (Figure 3) in most cases did not change significantly after the sterilization process. Only in the case of EtOH and e-beam sterilization, did the porosity value decrease significantly compared to nonsterile material.

#### 2.2.2. Chemical Analysis

**Figure 4:**
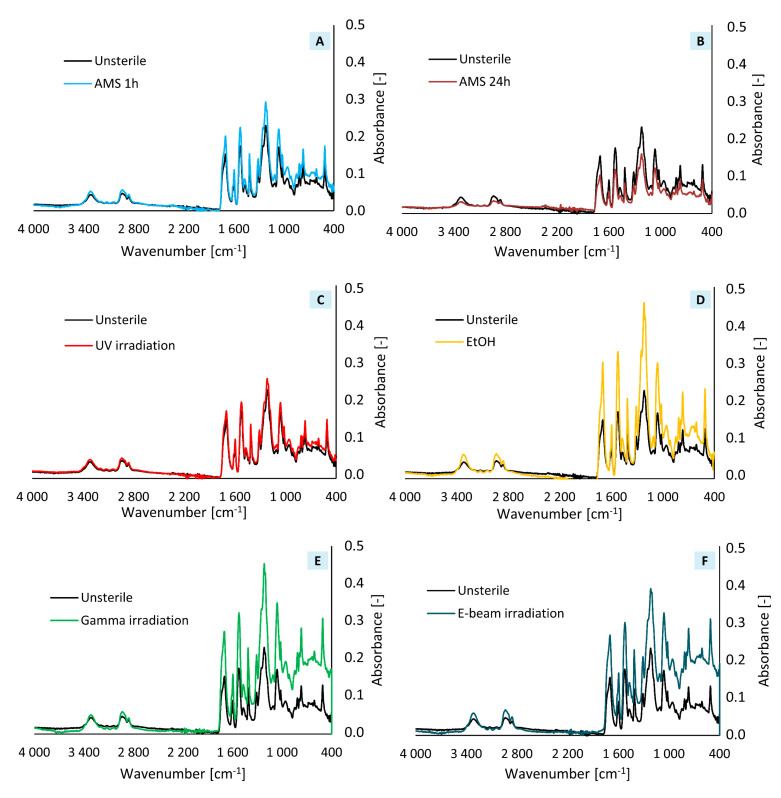
FTIR spectra (**A**) AMS 1 h, (**B**) AMS 24 h, (**C**) UV irradiation, (**D**) EtOH, (**E**) gamma irradiation, (**F**) e-beam irradiation in comparison to unsterile material.

The results of FTIR analysis are shown in Figure 4. According to the literature, high intensity polyurethane spectrum adsorption bands around 1700–1750 cm^−1^ correspond to the C=O (carbonyl) group, and those around 1100–1200 cm^−1^ corresponds to the C-O-C (ether) group. The moderate intensity absorption bands around 3300 cm^−1^ are associated with the N-H group and those around 2800–2900 cm^−1^ are associated with the C-H (methylene) group. The obtained FTIR spectra are typical for polyurethanes and consistent with literature data [27,28,29,30].

FTIR spectra obtained for AMS 1 h and UV-irradiation coincide with the spectrum obtained for the unsterile sample.

#### 2.2.3. Mechanical Tests Results

**Figure 5:**
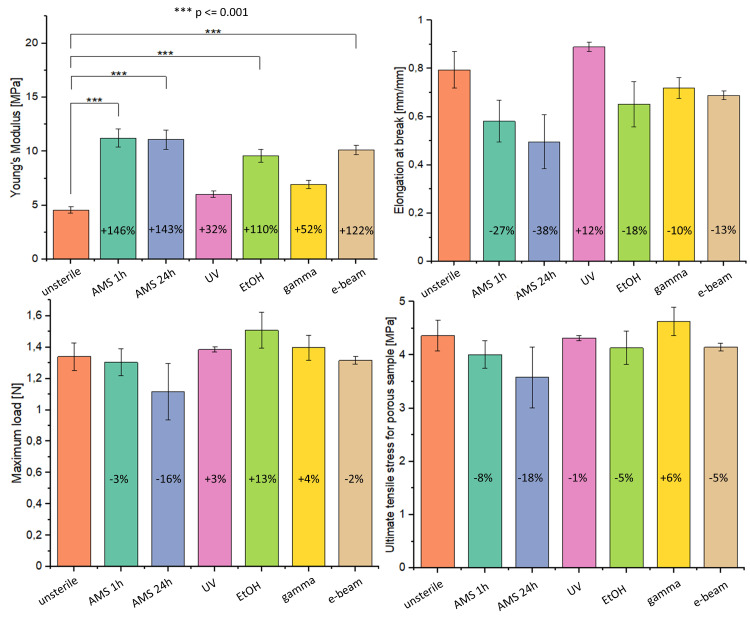
Mechanical properties of tested materials.

Mechanical tests results are presented in Figure 5. Material sterilization increases the value of Young’s modulus from 4.57 MPa for unsterile material up to about 10–11 MPa for material treated with AMS 1 h, AMS 24 h, EtOH, and E-beam. The values of Young’s modulus for UV- and gamma-irradiated samples did not differ significantly from the value for unsterilized samples.

No significant changes in ultimate tensile stress for porous sample value, maximum load value, and elongation at break value was observed after sterilization.

### 2.3. Cell Viability

#### 2.3.1. Cell Viability

**Figure 6:**
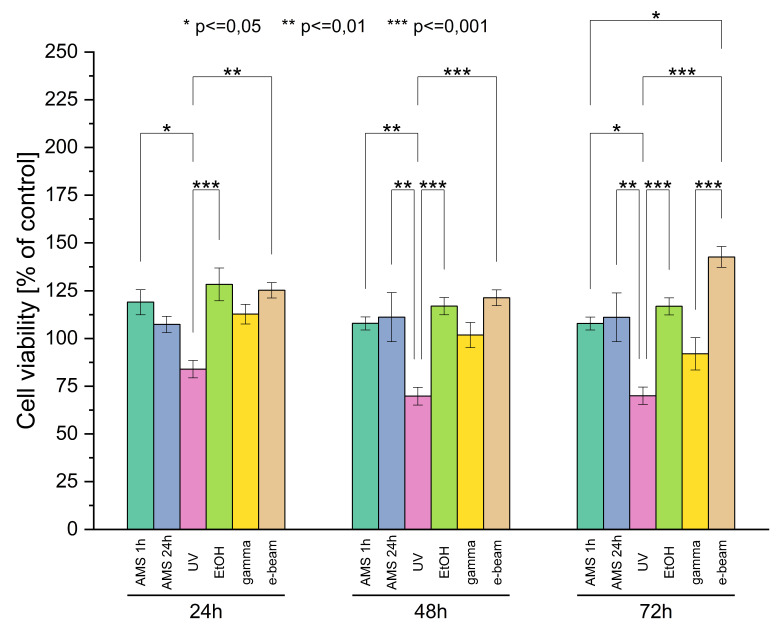
Cell viability test results.

The results of the alamar blue tests are presented in Figure 6. After 24 h of cell culture, the highest viability (about 130% of control) was observed for cells grown on EtOH sterilized materials. Slightly lower viability (about 120% of control) was observed for cells grown on materials treated with AMS 1 h and gamma irradiation. Cells grown on surfaces sterilized with AMS 24 h and surfaces radiated with gamma revealed viability close to control samples. The lowest viability (about 80% of control) revealed cells grown on UV-irradiated materials.

After 48 h of culture, cell viability did not change significantly compared to viability obtained after 24 h of culture. However, after 74 h of culture, a decrease in cell viability was observed for materials treated with UV and gamma radiation. Surprisingly, a meaningful increase in cell viability was observed for cells grown on e-beam-irradiated materials.

#### 2.3.2. Cell Adhesion

**Figure 7:**
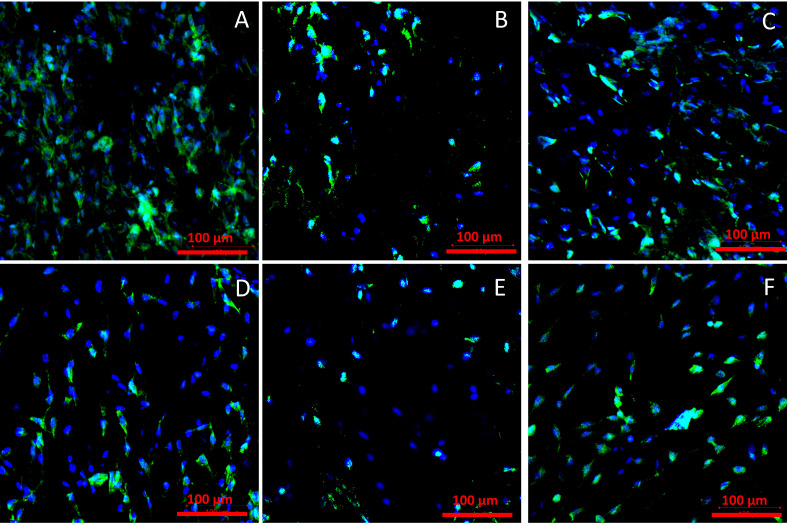
Materials’ surfaces after 72 h cell adhesion for (**A**) AMS 1 h, (**B**) AMS 24 h, (**C**) UV irradiation, (**D**) EtOH, (**E**) gamma irradiation, (**F**) e-beam irradiation. Cell nuclei stained with DAPI dye (blue) and actin stained with AlexaFluor488 dye (green).

Representations of cells adhered to the materials after 72 h of culture are presented in Figure 7.

Observation of the materials surfaces shows that the cell adhesion is similar for all materials. On each analyzed surface, cells presented proper morphology, with flattened cell body and with no apoptotic features. A slightly higher number of surface-adhered cells were observed for materials sterilized with AMS for 1 h and for materials sterilized with UV.

## 3. Discussion

In this work, we proposed five different techniques for sterilization of nanofibrous polyurethane scaffolds. Our aim was to achieve successful sterilization without a significant change in the morphology, chemical structure, and mechanical properties of the scaffolds.

Sterilization of scaffolds with an antimicrobial cocktail containing antibiotics (mostly mix of penicillin and streptomycin), and antimycotic (the most commonly used is amphotericin B) is a relatively novel technique. The advantages of this method certainly include simplicity and convenience. Antibiotics/antimycotics mechanism of action is based on binding to cell organelles and disturbing cell functions. The use of antibiotics allows the inactivation of most bacteria by disrupting processes such as DNA synthesis, synthesis of proteins, or cell wall components [3]. Streptomycin binds to a bacterial ribosome and inhibits protein synthesis [31]. Penicillin inhibits Gram-positive bacteria’s cell wall synthesis through binding to transpeptidase [32]. Amphotericin B binds to ergosterol in the cell membrane and ion channels of fungi through which protons and cations escape are formatted. Such depolarization leads to cell lysis [33]. Sterilization of polyurethane materials using the antimicrobial solution (AMS) has proven to be effective. During seven-day incubation, there were no signs of infections observed for all samples contacted with agar. The process of AMS sterilization did not affect surface topography. In addition, the structure of single fibers was not affected by AMS. However, a slight increase in average fiber diameter was observed. The value was higher for longer AMS incubation time (24 h) compared to the shorter incubation time (1 h). Similar results were reported by others—Braghirolli et al. reported changes in morphology for a nanofiber scaffold sterilized with AMS [12]. However, an increase in material rigidity was observed (significant increase of Young’s modulus and decrease of elongation at break values). The increase in Young’s modulus values was observed for both tested contact times (1 h and 24 h). Longer sterilization time influenced materials’ chemical structure changes. Other authors reported increased cellular adhesion for scaffolds sterilized with antimicrobial solutions for one hour and two hours [12]. Longer incubation time did not result in such an effect.

One of the concerns associated with UV sterilization is the change in the mechanical properties of the material, especially in the case of nanofibrous structures. For example, Duzyer et al. reported changes in Young’s modulus of nanofibrous polyethylene terephthalate (PET) after sterilization with UV [34]. However, the change was observed only for fibers produced from 15% wt. solution (average fiber diameter about 870 nm). For these structures, an increase in the average fiber diameter has also been shown. There were no significant changes in fiber diameter and Young’s modulus observed in the case of fibers produced from 10% wt. (average fiber diameter about 660 nm) and 20% wt. solutions (average fiber diameter about 2360 nm). In our study (where the average fiber diameter was about 595 nm), only a slight increase in the fiber diameter was observed (+3%). There was also a slight change in Young’s modulus value, but this was the smallest change among all sterilization techniques tested. It can be thus concluded that UV radiation did not change the morphology and mechanical properties of the fibers.

The second concern associated with UV radiation is its effectiveness, especially in the case of fiber materials with high porosity. UV treatment is considered as a disinfection method instead of sterilization. UV light is absorbed by microorganisms’ nucleic acids. Thereby, dimerization of pyrimidine molecules occurs and replication processes are inhibited [35]. Many authors reported successful sterilization of fibrous structures with UV. However, in our study, the UV sterilization turned out to be ineffective, which was confirmed by the agar diffusion test. The antibacterial effect of UV treatment depends on the time of exposure. However, prolonged UV exposure might result in strong changes in polymer properties, such as topography and chemical composition [15].

A 60–80% ethanol solution (EtOH) is a commonly used disinfection technique because it does not affect chemical and morphological scaffolds properties, is easily accessible, and is easy to use. However, EtOH solution in most cases needs to be combined with other sterilization techniques because hydrophilic viruses and bacterial spores are not susceptible to 60–80% ethanol solution [36]. High concentrations of EtOH solution (60–90%) cause denaturation of proteins and membrane damage which leads to metabolic processes disruption and cell lysis [37]. Effective sterilization is ensured by the combination of EtOH solution and UV radiation [38,39]. Treatment time also has a significant impact on its success. Selim et al. reported 5 min EtOH disinfection as ineffective for fibrous materials [11]. We reported that 20 min EtOH treatment is also an ineffective disinfection method for fibrous polyurethane material, which was confirmed by agar diffusion test. However, cell viability tests and cell adhesion observation showed no harmful effect of the material on the cells. One of the concerns associated with EtOH treatment is material shrinkage after sterilization. Gualandi et al. in their work observed that poly(L-lactic acid) (PLLA) fibrous materials after EtOH sterilization shrink and average fiber diameter increase. Higher EtOH concentration caused greater material shrinkage and increase in fiber size. They proved that material constrains prevent shrinkage and limit diameter changes [10]. Shrink effect was also observed in He et al. work [40]. Selim et al. in their work also observed a decrease in fiber diameter of about 50% [11]. In our research, the fiber diameter increased by about 15% after disinfection compared to the nonsterilized material. This was one of the biggest diameters change of all tested sterilization/disinfection methods.

Another concern associated with ethanol treatment is its influence on materials’ mechanical properties. Many authors reported a Young’s modulus value increase and a material tensile strength decrease after ethanol disinfection. However, these changes were always related to fiber diameter decrease [11,12,41]. In our work, we observed a significant increase of Young’s modulus value (+110% compared to the nonsterilized material) and a decrease of elongation at break value (−18%) which testifies to material rigidity increase. However, material tensile strength has not changed.

Gamma irradiation exposure might result in breaking polymer molecular chains into smaller fragments, which leads to change in material properties and its degradation. However, it is widely known that aromatic polymers, like polyurethanes, are resistant to high-energy radiation [42]. FTIR analysis of our gamma-treated samples did not show any additional peeks on FTIR spectra, thus sterilization did not affect materials chemical composition. Despite this we observed material color change after incubation with culture medium. Furthermore, we reported the biggest fiber diameter change (+16%) of all tested sterilization/disinfection methods (compared to the nonsterilized material). Marreco et al. in their work also observed chitosan-membrane color change after gamma sterilization. The authors reported that color change might be caused by the formation of supersaturations or color complexes, which lead to deterioration of mechanical properties and finally material degradation [9]. Bosworth et al. in their work reported that, despite the lack of visible changes in the fibers morphology immediately after sterilization, degradation of the poly(lactic-co-glycolic acid) (PLGA) membrane occurs much faster than the degradation of the non-sterile membrane [43]. A similar phenomenon was observed at work [11]. In the case of aliphatic polymers (e.g. polycaprolactone (PCL), PLLA, PLGA), many authors reported significant changes in Young’s modulus and tensile strength after sterilization [11,41,43,44,45]. We reported no statistically significant differences in materials’ mechanical properties after gamma sterilization. Thus, the resistance of polyurethanes to gamma irradiation is also confirmed by mechanical tests. During gamma sterilization, high-energy photons emitted by a radioactive ^60^Co source bombard material with microorganisms, which leads to electron displacement. Thereby, free radicals that breaking chemical bonds (including microbial DNA) are generated. As a consequence, all organisms present on the material are killed [46]. Although gamma irradiation has no influence on polyurethane scaffolds’ mechanical properties, it turned out to be an ineffective sterilization method, which was confirmed by agar diffusion and cell viability tests. Even though many authors reported gamma sterilization as an effective method for fibrous materials [11,43] the very rapid degradation of the material following sterilization makes this method unsuitable for materials for biomedical applications.

Electron beam (e-beam) irradiation similarly to gamma and UV irradiation might cause the degradation of the material, in particular the change of its mechanical properties. The mechanism of action is close enough to gamma irradiation, but it is distinguished by short exposition time of high electron energy [4]. Because of the lower photon energy in comparison to gamma irradiation, e-beam sterilization should have less impact on the physical and chemical material properties [3], which was confirmed in Cassan et al.’s work. The authors confirmed that the material with the lowest impact on PCL’s properties, out of gamma, X-ray, and e-beam irradiation, was e-beam irradiation [47]. Similarly to gamma sterilization, we did not observe any changes in the FTIR spectrum after e-beam treatment. Also, no change in fiber diameter was observed after sterilization. Other authors reported a significant decrease in fiber diameter of PLLA materials after e-beam irradiation [48]. However, we observed a significant change in Young’s modulus value (+122% compared to the nonsterilized material). Thus, e-beam irradiation caused an increase in material rigidity but did not change the material’s mechanical strength after sterilization. E-beam irradiation, like gamma irradiation, turned out to be an ineffective sterilization method, which was also confirmed by the agar diffusion test. Despite this, the cell viability test shows high cell activity after seven days of growth.

Not all sterilization methods are suitable for all types of material. In our work, we proved that immersion in an antibiotics/antimycotics cocktail might be a successful method for sterilization of fibrous polyurethane materials. Additionally, shorter incubation time (1 h) appears to be more appropriate due to the lower impact on material structure and its chemical properties.

## 4. Materials and Methods

### 4.1. Materials Fabrication

Fibrous materials were fabricated from polyurethane (ChronoFlex C75D, AdvanSource Biomaterials, Wilminghton, MA, USA) in the solution blow spinning (SBS) process. As a solvent 1,1,1,3,3,3-hexafluoroisopropanol (>99.0%, TCI Chemicals, Fukaya City, Japan) was used.

**Figure 8:**
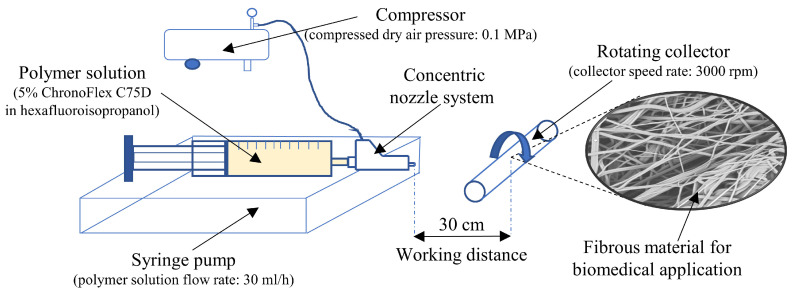
Solution blow spinning scheme.

Polymer solution in a concentration of 5% (*w/w*), prepared overnight, was supplied through the inner nozzle, at the same time, compressed air was supplied through the outer nozzle of the concentric nozzle system. Polyurethane solution was formed and dragged towards the collector by the high-speed compressed dry airflow. The concentric nozzle system is the main part of the SBS setup (Figure 8). Polymer solution flow rate was controlled by a syringe pump (KDS 100, KDS Scientific Inc., Holliston, MA, USA). Materials were prepared with a constant flow rate of 30 mL/h and at a constant air pressure of 0.1 MPa. The working distance between the collector and concentric nozzle system was 30 cm, the speed of the collector was 3000 rpm.

### 4.2. Sterilization Techniques

#### 4.2.1. Antimicrobial Solution (AMS)

Scaffolds were submerged in antimicrobial solution (1% (*v/v*), penicillin-streptomycin (100 U/mL, Gibco^TM^, ThermoFisher Scientific, Waltham, MA, USA) and 0,1% (*v/v*) amphotericin B ((Gibco^TM^, ThermoFisher Scientific, Waltham, MA, USA) diluted in sterile PBS) at 4 °C for 1 h (AMS 1 h) or 24 h (AMS 24 h). Next, samples were washed in sterile PBS on a plate shaker for five minutes, repeated five times. Finally, samples were dried or used directly for testing.

#### 4.2.2. Ethanol Solution

Materials were immersed in 70% (*v/v*) ethanol solution at 20 °C for 20 min. Next, samples were washed five times in sterile phosphate buffered saline (PBS) on a plate shaker for five minutes each time. Finally, samples were dried or used directly for testing.

#### 4.2.3. UV Irradiation

Flat materials were UV irradiated for 30 min on each side. Materials were located on the countertop in the laminar flow hood (Msc-Advantage, Thermo Scientific, Waltham, MA, USA) as a UV source, a lamp from the laminar hood was used. Samples were used for testing directly after sterilization.

#### 4.2.4. Gamma Irradiation

Materials were irradiated at room temperature with a standard dose of 25 kGy and dose rate 2.44 kGy/h. ^60^Co gamma-ray source was used. Gamma irradiation sterilization was carried out at Institute of Nuclear Chemistry and Technology, Warsaw, Poland.

#### 4.2.5. E-Beam Irradiation

E-beam sterilization procedure with a dose of 25 kGy was carried out using 10 MeV, 10 kV linear electron accelerator Elektronika 10/10 with a dose rate 5 × 10^3^ Gy/s in Institute of Nuclear Chemistry and Technology, Warsaw, Poland. Delivered dose was confirmed using B3 radiochromic foil dosimeter.

### 4.3. Evaluation of Sterilization Efficiency

#### 4.3.1. Contact with Cell Culture Medium

Sterile round-shaped samples (*n* = three per sterilization method) together with the positive control (*n* = three unsterilized samples) were placed in 48-well plates and incubated with Dulbecco’s Modified Eagle Medium (DMEM) supplemented with 10% FBS (Gibco^TM^, ThermoFisher Scientific, Waltham, MA, USA) at 37 °C in a 5% CO_2_ humidified atmosphere. After 24 h, 48 h, 72 h, and seven days of incubation, the condition of the medium was evaluated using an inverted optical microscope (Olympus, Hamburg, Germany).

#### 4.3.2. Agar Diffusion

The evaluation of sterilization efficiency was carried out using agar diffusion method. LB broth base (Lennox L Broth Base) containing peptone, yeasts extract and NaCl (Thermo Fisher Scientific) together with bacteriological agar (BTL) was dissolved in distilled water to prepare agar medium (1% *m/v* pepton, 0.5% *m/v* yeasts extract, 0.5 % *m/v* NaCl, 0.15% *m/v* bacteriological agar). Following steam sterilization under 0.1 MPa pressure at 121 °C for 15 min using steam sterilizer (SMS, ASSVE/46 W), hot agar medium was poured into 6 cm-diameter Petri dishes. Petri dishes were allowed to cool down so that the agar solidified. Then, round-shaped material samples (*n* = three for every type of sterilized material and unsterilized material (positive control) with diameter of 8 mm were put on agar medium in the center of Petri dishes and incubated at 37 °C. As a positive control, unsterile materials samples were used (*n* = 3). Additionally, negative control (*n* = 3 Petri dishes with agar with no materials) was prepared. After 24, 48 and 72 h of incubation, the condition of the agar was visually evaluated.

### 4.4. Materials Characterization

#### 4.4.1. Surface Morphology

Dry, sterile square-shaped samples (*n* = two per method) were stuck to stubs using conductive carbon tape and coated with a 15 mm layer of gold using a sputter coater (K550 Emitech, Quorum Technologies, Laughton, East Sussex., United Kingdom). Surface morphology was examined using scanning electron microscopy (SEM, Phenom G1, Phenom World, Eindhoven, Netherlands). Samples were photographed (*n* = 10 images per sample) in ×600 and ×1000 magnification.

SEM images were used for fiber diameter measurement. *n* = 100 fiber diameter was measured using Fiji software [49] per each sterilized and unsterile material. Distribution of fiber diameters before and after sterilization was presented in the form of a box chart.

#### 4.4.2. Chemical Analysis

Fourier transform infrared spectroscopy (FTIR) was used to define changes in a surface chemical structure after sterilization. Sterile and unsterile dry round-shaped samples (*n* = two per each method) were analyzed using Nicolet™ 6700 spectrometer (Thermo Scientific, Waltham, Massachusetts, USA) in the wavenumber range between 400 and 4000 cm^−1^. Thirty-two scans for every sample were done. Results are presented as FTIR spectra.

#### 4.4.3. Mechanical Properties

Sterile flat samples with dimensions 70 mm × 5 mm (*n* = five per each sterilization method) were subjected to a mechanical test using Instron 3345 testing machine. Samples were placed inside the sample holder and stretched at the rate of 5 mm/min following ASTM D638-02a [50]. The Young’s modulus, elongation at break, and ultimate tensile stress for flat and porous sample results were automatically calculated by computer software. Results are determined as mean value ± SD and percentage change in the value relative to control (unsterile material).

Ultimate tensile stress for porous samples (σ_por_) was determined using the Formula (1):

(1) $${\;\sigma }_{\mathrm{por}}=\frac{F_{\max}}{A\left(1-\varepsilon \right)}$$

Young’s modulus (E) was determined using the Formula (2):

(2) $$E=\frac{\sigma_{\mathrm{por}}}{L}$$

where:

F_max_—maximum force acting on the sample [N]
A—cross-section area [m^2^]
ε—sample porosity [-]
L—sample deformation [m]

#### 4.4.4. Materials’ Porosity

Materials’ porosity (ε) was determined using the Formula (3):

(3) $$\varepsilon =1-\frac{\rho_{\mathrm{sample}}}{\rho_{\mathrm{PU}}}$$

where:

ρ_PU_—ChronoFlex^®^C75D density [g/cm^3^]
  (4) $${\;\rho }_{\mathrm{PU}}=1.2\frac{g}{{\mathrm{cm}}^{3}}$$
ρ_sample_—sample density [g/cm^3^] [51]
  (5) $$\rho_{\mathrm{sample}}=\frac{m}{A*\delta }$$

where:

m—sample weight [g]
A—sample area [g/cm^3^]
δ—sample thickness [cm]

Three flat square samples with dimensions 10 mm × 10 mm were used to determine materials’ porosity. An analytical balance was used to determine samples weight and surface section SEM images in ×300 magnification were used to sample thickness measurements. Fifteen thickness measurements were done per each sample. Results are determined as mean value ± SD and percentage change in the value relative to control (unsterile material).

### 4.5. Cell Viability

#### 4.5.1. Cell Culture Preparation

L929 immortalized mouse fibroblast cell line was cultured in DMEM with phenol red supplemented with 10% fetal bovine serum (FBS) (Gibco^TM^, ThermoFisher Scientific, Waltham, MA, USA) and 2 mM L-glutamine (Gibco^TM^, ThermoFisher Scientific, Waltham, MA, USA) at 37 °C in a 5% CO_2_ humidified atmosphere. DMEM medium was changed every three to four days. Cells were subcultured with 90% confluence ratio by trypsinization using 0.05% trypsin/0.02% EDTA solution with phenol red (Gibco^TM^ ThermoFisher Scientific, Waltham, Massachusetts, USA). Cells were counted using a hemocytometer (Sigma-Aldrich, Merck KGaA, Darmstadt, Germany).

#### 4.5.2. Cell Viability

Cell viability was tested using the alamar blue assay (ThermoFisher Scientific, Waltham, MA, USA) according to the manufacturer’s protocol. Sterile materials were placed in 48-well plates, mounted with inserts, and incubated in 500 ul DMEM for one hour at 37 °C in a 5% CO_2_ humidified atmosphere. Following incubation, L929 cells were seeded on the material and blank wells with no material (control) at the seeding density 1 × 10^5^ cells/mL and cultured for 24 h. After 24, 48, and 72 h, the medium was removed from wells and 500 µL of alamar blue working solution (alamar blue reagent 10× diluted in fresh DMEM without phenol red) was added (500 µL/well). Materials were incubated for 4 h at 37 °C in a 5% CO_2_ humidified atmosphere (protected from light). Following incubation, 100 µL of alamar blue solution was transferred in triplicate to a 96-well plate (black) and fluorescence (Ex. = 550 nm, Em. = 590 nm) was measured. Samples were washed with PBS (three times for five minutes on a plate shaker), incubated with fresh DMEM, and used for further viability measurements. Viability results are presented as the percentage of positive control according to the Formula (6):

(6) $$V=\frac{{\mathrm{FI}}_{\mathrm{sample}}}{{\mathrm{FI}}_{\mathrm{control}24\;h}}*100\%$$

where:

V—cells viability [%]
FI_sample_—sample’s fluorescent intensity [-]
FI_control24h_—control’s fluorescent intensity after 24 h of culture [-]

#### 4.5.3. Cell Adhesion

Cell adhesion was observed using a confocal microscope equipped with ZEISS ZEN software (Zen 2, ZEISS, Oberkochen, Germany). After 72 h of culture, samples were washed with PBS (four times for five minutes on a plate shaker) and fixed with 4% (*w/v*) paraformaldehyde (500 µL/well, (Sigma-Aldrich, Merck KGaA, Darmstadt, Germany). Plates were incubated at 4 °C for 24 h and washed with PBS (three times for five minutes on a plate shaker). Then cells were permeabilized by adding 500 µL/well of 0.2% Triton X-100 for eight minutes. Materials were washed with PBS (four times for five minutes on a plate shaker). Next, materials were washed with PBS (four times for five minutes on a plate shaker) and incubated with 2.5% AlexaFluor488 Phalloidin in PBS solution (ThermoFisher Scientific, Waltham, MA, USA) at room temperature for 1 h in the dark. Then materials were washed with PBS (four times for five minutes on a plate shaker) and incubated with 300 nM DAPI (300 µL/well, ThermoFisher Scientific, Waltham, MA, USA) at room temperature for six minutes in the dark. Finally, samples were washed with PBS (four times for five minutes on a plate shaker), placed on microscope slides with a drop of glue (ProLong Diamond Antifade Mountant, Invitrogen, ThermoFisher Scientific, Waltham, MA, USA), and covered with cover slides. Following described preparation samples (three for each sterilization method) were observed using confocal microscope. Cell nuclei were stained with DAPI dye and cell cytoplasm with AlexaFluor488 dye. Six images at ×20 magnification were taken for each type of sample.

### 4.6. Statistical Analysis

The results of the measurements were expressed as means ± SD. Statistical significance of differences was analyzed using a single-factor or two-factor analysis of variance (ANOVA) for *p* < 0.05 with post hoc Tukey’s test (OriginPRO 2020b, OriginLab Corporation, Northampton, MA, USA).

## 5. Conclusions

Effective sterilization of polymeric fibrous materials can be challenging due to the high porosity of the material. At the same time, the structure of the nanofibrous material can be quite easily damaged, resulting in a change in the properties of the material. The aim of the work was to evaluate classical sterilization methods (radiation, ethanol immersion) as well as a relatively new and less commonly used method (antimicrobial solution).

Our studies have shown that radiation methods as well as ethanol immersion did not guarantee high sterilization efficiency. Application of those techniques also resulted in a statistically significant change in porosity, the Young’s modulus, as well as a decrease in the viability of cells cultured in contact with the material.

As an alternative sterilization method, we proposed immersion in antimicrobial solution. We proved that this technique allowed us to achieve high efficiency of the sterilization process. At the same time it is mild to the nanofibrous material and does not cause a significant change in materials properties.

## Figures and Tables

**Table 1:** Efficiency of different sterilization techniques.

	Unsterile	AMS 1 h	AMS 24 h	UV Irradiation	EtOH	Gamma Irradiation	E-Beam Irradiation
Material morphology (visual inspection)	−	−	−	−	−	+ *	−
Contact with cell culture medium	−	−	−	−	−	−	−
Contact with Agar	+	−	−	+	+	+	+

“+” indicates contamination or macroscopic changes of the material, “−“ indicates no visible changes of the material or medium, * the change was observed after incubation with culture medium.
